# Use of transient elastography to assess hepatic steatosis and fibrosis in patients with juvenile idiopathic arthritis during methotrexate treatment

**DOI:** 10.1007/s10067-023-06835-x

**Published:** 2023-12-08

**Authors:** Chayakamon Niyasom, Sirisucha Soponkanaporn, Soamarat Vilaiyuk, Chatmanee Lertudomphonwanit, Songpon Getsuwan, Pornthep Tanpawpong, Piyaporn Kaewduang, Abhasnee Sobhonslidsuk

**Affiliations:** 1 Division of Rheumatology, Department of Pediatrics, Faculty of Medicine Ramathibodi Hospital, Mahidol University, Bangkok, Thailand; 2 Division of Gastroenterology, Department of Pediatrics, Faculty of Medicine Ramathibodi Hospital, Mahidol University, Bangkok, Thailand; 3 Division of Gastroenterology and Hepatology, Department of Medicine, Faculty of Medicine Ramathibodi Hospital, Mahidol University, Bangkok, Thailand

**Keywords:** Hepatic fibrosis, Hepatic steatosis, Juvenile idiopathic arthritis, Methotrexate, Transient elastography

## Abstract

**Objectives:**

This study aimed to assess the prevalence and identify predictors of hepatic steatosis and fibrosis in patients with juvenile idiopathic arthritis (JIA) during methotrexate treatment.

**Method:**

This cross-sectional study included JIA patients who had received methotrexate for > 1 year. Laboratory data including liver chemistry and lipid profiles were collected. Liver stiffness measurements (LSM) and controlled attenuation parameters (CAP) were determined by transient elastography. Significant hepatic fibrosis was defined as LSM > 7 kilopascal (kPa), and hepatic steatosis was defined as CAP > 225 decibel/meter (dB/m). Logistic regression analysis was performed to identify predictors associated with hepatic steatosis and fibrosis.

**Results:**

Of 60 patients, 66.7% were female, and the median age (IQR) was 12.8 (10.6–15.0) years. The median duration of methotrexate usage (IQR) was 45 (22–85) months, and the median cumulative dose of methotrexate (IQR) was 3768 (1806–6466) mg. The median LSM (IQR) and CAP (IQR) were 4.1 (3.4–4.6) kPa and 191.0 (170.3–223.8) dB/m, respectively. No patients had transient elastography-defined hepatic fibrosis, whereas 21.7% had hepatic steatosis. A body mass index *Z*-score > 1 (OR 5.71 [95%CI 1.31–24.98], *p* = 0.021) and higher cumulative dose of methotrexate (OR 1.02 [95%CI 1.00–1.04], *p* = 0.041) were associated with hepatic steatosis, whereas the cumulative dose of steroids was not (OR 1.00 [95%CI 1.00–1.01], *p* = 0.097).

**Conclusions:**

Hepatic steatosis is common among JIA patients receiving methotrexate, but none had transient elastography-defined hepatic fibrosis. Overweight/obese JIA adolescents and patients with a high cumulative dose of methotrexate are at risk for hepatic steatosis.

**Table:** 

**Key Points** •*Long-term low-dose methotrexate usage and the concomitant use of other DMARDs did not increase the risk of hepatic fibrosis in JIA patients.* •*The prevalence of hepatic steatosis in JIA patients receiving methotrexate was higher than in a healthy pediatric population.* •*Overweight/obesity and a higher cumulative dose of methotrexate were predictors of hepatic steatosis.*

## Introduction

Juvenile idiopathic arthritis (JIA) is a heterogeneous group of chronic inflammatory arthropathies with unknown etiology occurring before the age of 16 years [1]. Recent guidelines for the treatment of JIA have been developed according to the different clinical phenotypes of JIA [2, 3]. Methotrexate is recommended as a first-line disease-modifying antirheumatic drug (DMARD) for JIA. Although most JIA patients can tolerate methotrexate, its long-term usage is a risk factor for liver-related adverse events such as elevated liver enzymes, hepatic steatosis, fibrosis, and cirrhosis in adult patients with rheumatoid arthritis (RA) [4].

The gold standard for the diagnosis of hepatic steatosis and fibrosis is liver biopsy [5]. Because it is an invasive method and has an inherent risk of serious complications, liver biopsy cannot be performed routinely in all patients with JIA. Various non-invasive techniques, such as the measurement of liver enzymes, ultrasonography, and magnetic resonance imaging, have been utilized to evaluate liver-related adverse events [6, 7]. Conventional liver chemistry had a poor correlation with the occurrence of hepatic fibrosis [8]. Ultrasound elastography, also called transient elastography (TE), or FibroScan® with a controlled attenuation parameter (CAP) is a novel technique that has been validated to detect hepatic fibrosis and steatosis [9]. Most studies of TE evaluated adult patients with psoriasis, psoriatic arthritis, or RA receiving methotrexate therapy [10, 11]. In pediatric patients with chronic liver disease, including chronic hepatitis C virus infection, autoimmune hepatitis, and Wilson disease, TE detected hepatic fibrosis with a sensitivity and specificity ranging from 81.4 to 100% and 75.0 to 97.2%, respectively [12]. However, there are limited data related to the evaluation of hepatic fibrosis and steatosis in pediatric patients with JIA receiving methotrexate treatment using TE with CAP.

The purpose of this study was to evaluate the prevalence of hepatic steatosis and fibrosis using TE with CAP in methotrexate-treated patients with JIA. In addition, we aimed to identify predictors associated with the development of TE-defined hepatic fibrosis and steatosis.

## Methods

### Design and study population

This cross-sectional study was conducted at the Faculty of Medicine Ramathibodi Hospital, Mahidol University, a tertiary-care medical center in Bangkok, Thailand, between April 2021 and January 2022. We recruited all patients with JIA diagnosed using the International League of Associations for Rheumatology criteria [1]. Inclusion criteria were patients with JIA from 1 to 18 years old who had received methotrexate for more than 1 year. Patients with chronic liver diseases such as hepatitis B or C infection or autoimmune hepatitis, or who used alcohol, or who had poor adherence to medical treatment were excluded. All patients received the same standard treatments including methotrexate at 6–25 mg/m^2^/week (maximum dose 25 mg/week) with 1.25 mg/day of daily folic acid supplementation [2, 3]. Concomitant medication at the enrollment is demonstrated in Table 1.

**Table 1:** Baseline characteristics and laboratory data of JIA patients at enrollment

Parameter	*N* = 60
Female, *n* (%)	40 (66.7)
Age (years)	12.8 (10.6–15.0)
BMI *Z*-score	− 0.01 (− 0.89–0.88)
Disease duration (years)	4.2 (2.1–7.8)
JIA subtype, *n* (%)	
Systemic arthritis	17 (28.3)
Enthesitis-related arthritis	11 (18.3)
Polyarticular JIA (RF +)	15 (25.0)
Polyarticular JIA (RF −)	7 (11.7)
Oligoarticular JIA	9 (15.0)
Undifferentiated JIA	1 (1.7)
JADAS-27 score	6 (2.2–9.3)
Current medication information	
Steroids, *n* (%)	33 (55.0)
Sulfasalazine, *n* (%)	41 (68.3)
Azathioprine, *n* (%)	13 (21.7)
Cyclosporin A, *n* (%)	7 (11.7)
Leflunomide, *n* (%)	6 (10.0)
Mycophenolate mofetil, *n* (%)	1 (1.7)
Biologic DMARDs, *n* (%)	22 (36.7)
Etanercept	12 (20.0)
Tocilizumab	9 (15.0)
Adalimumab	1 (1.7)
Duration of methotrexate use (months)	45.0 (22.2–84.5)
Cumulative dose of methotrexate (mg)	3767.5 (1806.0–6466.0)
Duration of steroid use (months)	5.3 (0.0–28.5)
Cumulative dose of steroids (mg)	1562.5 (0.0–7694.4)
Laboratory (median, IQR)	
AST (U/L)	24.0 (20.0–30.8)
ALT (U/L)	18.5 (13.0–27.3)
GGT (U/L)	16.5 (13.3–21.8)
ESR (mm/h)	13.0 (6.0–28.0)
CRP (mg/dL)	1.9 (0.0–6.5)
Triglyceride (mg/dL)	77.5 (57.0–110.3)
Cholesterol (mg/dL)	174.0 (154.8–200.8)
HDL cholesterol (mg/dL)	52.0 (44.3–66.5)
LDL cholesterol (mg/dL)	119.0 (89.5–144.0)

Data expressed as the median (IQR) unless otherwise indicated

*ALT* alanine transaminase, *AST* aspartate transaminase, *BMI* body mass index, *CRP* c-reactive protein, *DMARDs* disease-modifying antirheumatic drugs, *ESR* erythrocyte sedimentation rate, *GGT* gamma-glutamyl transferase, *HDL* high-density lipoprotein, *IQR* interquartile range, *JADAS* Juvenile Arthritis Disease Activity Score, *JIA* juvenile idiopathic arthritis, *LDL* low-density lipoprotein, *RF* rheumatoid factor

### Clinical parameters

Demographic data and clinical characteristics were obtained from medical records including sex, age, disease duration, subtype of JIA, disease activity, and body mass index (BMI) *Z*-score at enrollment. Disease activity was assessed using the Juvenile Arthritis Disease Activity Score-27 (JADAS-27) [13], which was calculated by the summation of the scores of four values: physician’s global assessment of disease activity, parents’ or patients’ global assessment of well-being, erythrocyte sedimentation rate (ESR) (calculated using the formula: ESR (mm/h) − 20/10), and a count of active joints. JADAS-27 scores ranged from 0 to 57, with higher scores indicating more active disease. The cutoff score for high disease activity was ≥ 6 [14]. The BMI *Z*-score was calculated based on the World Health Organization growth charts [15]. Patients with a BMI *Z*-score > 1 to 1.99 were classified as overweight and those with a BMI *Z*-score ≥ 2 as obese [15]. Data related to medications including cumulative dose and duration of methotrexate and steroids, route of methotrexate therapy, use of other conventional DMARDs, biologic DMARDs, nonsteroidal anti-inflammatory drugs (NSAIDs), and acetaminophen were recorded. Cumulative doses of methotrexate and steroids were calculated from the start of medication until the time of enrollment.

Laboratory data including complete blood count, ESR, C-reactive protein, liver enzymes, lipid profile, blood urea nitrogen, and creatinine were collected at enrollment. Liver enzymes were routinely measured every 2–12 weeks. Patients who were commencing methotrexate or stepping up the dose of methotrexate had liver enzymes measured 2–4 weeks thereafter, and patients who received a stable dose of methotrexate had liver enzymes measured every 8–12 weeks. Abnormal serum aspartate aminotransferase (ALT) was defined as ALT > 26 U/L [16]. The frequencies and duration of abnormal ALT levels and the maximum level of ALT were recorded. If serum levels of ALT increased > 2 times the upper limit of normal (ULN), the dose of methotrexate was reduced. Methotrexate was discontinued if the serum ALT was > 3 times the ULN and then restarted upon the normalization of ALT. Patients with persistent abnormal ALT levels underwent abdominal ultrasonography, viral hepatitis serologic profiles, and measurement of autoantibodies associated with autoimmune hepatitis. The cutoff levels for high total cholesterol and low-density lipoprotein (LDL)-cholesterol were > 199 and > 129 mg/dL, respectively. High levels of triglyceride were > 99 and 129 mg/dL in patients aged < 10 years and 10–19 years, respectively. The cutoff for a low level of high-density lipoprotein (HDL)-cholesterol was < 40 mg/dL [17].

### Outcome measures

Hepatic fibrosis and steatosis were evaluated using TE (FibroScan®, EchoSens, Paris, France) at enrollment. After at least 4 h of fasting, patients underwent TE by an experienced practitioner who was blinded to patient data. TE is a non-invasive ultrasound-based elastography technique consisting of a 3.5 MHz transducer probe. The probe transmits an elastic shear wave through the underlying liver parenchyma, and the measurement of liver stiffness is reported as the liver stiffness measurement (LSM) in kilopascals (kPa). A LSM value > 7 kPa indicates significant liver fibrosis [10, 18]. Because there was no cutoff value of LSM in patients with JIA, the cutoff of LSM is derived from studies in adults with RA and psoriasis. TE also measured CAP, which was used to assess hepatic steatosis. A CAP value of > 225 decibel/meter (dB/m) indicates hepatic steatosis [19].

### Statistical analysis

Data analysis was conducted using IBM SPSS Statistics for Windows, Version 28.0. Armonk, NY. Descriptive statistics were summarized as a number with percentages and median with interquartile range (IQR). The comparison of categorical and continuous variables was performed using Fisher’s exact test and the Mann–Whitney *U*-test, respectively. Correlation analysis was performed by Spearman’s correlation test. Logistic regression analysis was used to evaluate potential predictors of hepatic fibrosis and steatosis. The strength of predictors was estimated by odds ratio (OR) and 95% confidence intervals (CI). *p-*values < 0.05 were considered statistically significant.

## Results

### Baseline characteristics

Of the 60 patients with JIA, 40 (66.7%) were female, and the median age (IQR) was 12.8 (10.6–15.0) years. The median disease duration (IQR) was 4.2 (2.1–7.8) years, and 46 patients (76.7%) had a disease duration of > 2 years. The most common JIA subtype was systemic JIA (28.3%), followed by rheumatoid factor-positive polyarthritis (25.0%), and enthesitis-related arthritis (18.3%). Thirty-one patients (51.7%) had high disease activity. All patients were treated with methotrexate, folic acid, and NSAIDs. About half of the patients received steroids. Forty-three patients (71.7%) received oral methotrexate therapy, and the rest received subcutaneous methotrexate. The median cumulative dose and duration of methotrexate were 3767.5 (1806.0–6466.0) mg and 45.0 (22.2–84.5) months, respectively. About one-third of patients (*n* = 22) were treated with biologic DMARDs with etanercept being the most common drug. Seven patients (11.7%) were overweight, and 5 patients (8.3%) were obese. Seventeen patients (28.3%) had hypercholesterolemia, and 21 patients (35%) had high LDL cholesterol. The baseline characteristics and laboratory data of the patients are shown in Table 1.

### Hepatic assessment in patients with JIA during methotrexate treatment

All 60 patients had normal liver enzymes at enrollment. During the course of methotrexate treatment, 60% showed elevated transaminases. Among these patients, 28% had persistent elevated transaminases for > 6 months, and 22% had transaminases > 3 times the ULN. The methotrexate dosage was reduced in 13 patients. Methotrexate was withheld from 20 patients and re-prescribed when their transaminase levels returned to normal.

The median LSM was 4.1 (3.4–4.6) kPa, and no patients had TE-defined hepatic fibrosis. The median CAP was 191.0 (170.3–223.8) dB/m. Thirteen patients (21.7%) were categorized with hepatic steatosis.

### Correlation of LSM values with patients’ clinical characteristics and laboratory data

LSM values had a significant correlation with age (*r*^2^ = 0.263, *p* = 0.042). The cumulative dose of methotrexate, duration of methotrexate usage, BMI *Z*-score, JADAS-27, ALT, cholesterol, and LDL-cholesterol did not have a significant correlation with the LSM values (Table 2). To investigate the effect of the cumulative dose of methotrexate on LSM values further, the median LSM values were calculated for the two groups stratified by the cumulative methotrexate dosage (< 4000 mg and ≥ 4000 mg), according to a previous study [20]. The median LSM values were not significantly different between the groups (cumulative methotrexate dose < 4000 mg, 4.10 kPa, 3.30–4.55; cumulative methotrexate dose ≥ 4000 mg, 4.25 kPa, 3.78–4.60) (*p* = 0.27). Multiple linear regression analysis demonstrated that only age was significantly associated with the LSM values (standardized *β* = 0.348, 95%CI 0.007–0.158, *p* = 0.032).

**Table 2:** Correlation of liver stiffness measurement values with clinical and laboratory parameters

Parameter	Correlation coefficient (*r*^2^)	*p*-value
Age	0.263	0.042
JADAS-27 score	− 0.024	0.855
BMI *Z*-score	− 0.097	0.462
Cumulative dose of methotrexate (mg)	0.143	0.276
Duration of methotrexate use (months)	0.045	0.735
Cumulative dose of steroid (mg)	− 0.101	0.577
Duration of steroid use (months)	− 0.206	0.249
ALT (U/L)	0.045	0.732
Cholesterol (mg/dL)	0.057	0.664
LDL cholesterol (mg/dL)	0.077	0.559

*ALT* alanine transaminase, *BMI* body mass index, *JADAS* Juvenile Arthritis Disease Activity Score, *LDL* low-density lipoprotein

### Comparisons of clinical characteristics and laboratory data between JIA patients with and without hepatic steatosis during methotrexate treatment

There was no significant difference in the sex, age, JIA subtype, and disease activity scores between patients with and without hepatic steatosis (Table 3). The disease duration in patients with steatosis (median 6.6 [IQR 3.9–9.6] years) was significantly longer than in patients without steatosis (median 3.7 [IQR 1.6–7.4 years]) (*p* = 0.045). There was a significantly higher percentage of overweight/obesity (46.2%) in patients with steatosis compared with patients without steatosis (12.8%) (*p* = 0.015). Patients with steatosis had a positive history of elevated transaminase levels that were significantly higher than those in patients without steatosis (84.6 vs. 53.2%, *p* = 0.038). Among 13 patients with TE-defined hepatic steatosis, there were only five patients (38%) who had abnormal ALT, and none of them had ALT > 3 times the upper limit of normal. However, the median levels of ALT and gamma-glutamyl transferase at enrollment were not significantly different between patients with and without steatosis. The median serum cholesterol and LDL cholesterol levels in patients with steatosis tended to be higher than in patients without steatosis but were not significant.

**Table 3:** Clinical characteristics and laboratory data between JIA patients with hepatic steatosis and without hepatic steatosis

Parameter	Steatosis (*N* = 13)	Non-steatosis (*N* = 47)	*p*-value
Female, *n* (%)	8 (61.5)	32 (68.1)	0.448
Age (years)	14.2 (12.8–17.3)	12.3 (9.5–14.4)	0.439
BMI *Z*-score	0.92 (0.21–1.76)	− 0.40 (− 1.04–0.39)	*0.003*
Patients with BMI *Z*-score > 1, *n* (%)	6 (46.2)	6 (12.8)	*0.015*
Disease duration (years)	6.6 (3.9–9.6)	3.7 (1.6–7.4)	*0.045*
Systemic JIA, *n* (%)	5 (38.5)	12 (25.5)	0.488
JADAS-27 score	6.0 (3.0–8.4)	6.0 (2.0–10.4)	0.474
Medication information			
Duration of methotrexate use (months)	64.8 (36.2–107.5)	42.6 (17.4–83.3)	0.106
Cumulative dose of methotrexate (mg)	5405.0 (2760.0–9761.2)	3370.0 (1437.9–5614.0)	*0.017*
Steroids duration (months)	7.2 (0.0–53.0)	4.7 (0.0–23.7)	0.262
Steroids cumulative dose (mg)	1732.5 (0.0–26,657.0)	1410.0 (0.0–6545.0)	0.069
Concomitant use of sulfasalazine, *n* (%)	7 (53.8)	34 (72.3)	0.312
Concomitant use of azathioprine, *n* (%)	3 (23.1)	10 (21.3)	0.999
Concomitant use of leflunomide, *n* (%)	1 (7.7)	5 (10.6)	0.999
Concomitant use of biologic DMARDs, *n* (%)	6 (46.2)	16 (34.0)	0.520
Laboratory (*n*, %)			
Positive history of elevated transaminases	11 (84.6)	25 (53.2)	*0.038*
Elevated transaminases > 3 times of ULN	2 (18.2)	6 (24.0)	0.112
Persistent of elevated transaminases			0.082
< 3 months	4 (36.3)	14 (56.0)	
≥ 3 to < 6 months	4 (36.3)	4 (16.0)	
≥ 6 months	3 (27.4)	7 (28.0)	
ALT (U/L)	25.0 (12.0–36.5)	17.0 (11.0–27.0)	0.235
GGT (U/L)	20.0 (14.0–31.5)	16.0 (12.0–21.0)	0.061
LSM (kPa)	4.2 (3.5–4.5)	4.1 (3.4–4.6)	0.958
Lipid profile level			
Cholesterol (mg/dL)	182.0 (160.0–211.5)	173.0 (152.0–200.0)	0.351
LDL cholesterol (mg/dL)	125.0 (90.0–163.0)	112.0 (89.0–134.0)	0.276
HDL cholesterol (mg/dL)	53.0 (43.0–61.0)	52.0 (45.0–68.0)	0.491
Triglyceride (mg/dL)	81.0 (59.5–132.5)	76.0 (54.0–108.0)	0.344

*ALT* alanine transaminase, *BMI* body mass index, *DMARDs* disease-modifying antirheumatic drugs, *GGT* gamma-glutamyl transferase, *HDL* high-density lipoprotein, *IQR* interquartile range, *JADAS* Juvenile Arthritis Disease Activity Score, *JIA juvenile idiopathic arthritis,* *LDL* low-density lipoprotein, *LSM* liver stiffness measurement, *ULN* upper limit of normal

The median cumulative dose of methotrexate in patients with steatosis was significantly higher (5405.0 mg, IQR 2760.0–9761.2) than in patients without steatosis (3370.0 mg, IQR 1437.9–5614.0) (*p* = 0.017). The cumulative dose of methotrexate and steroid per weight was not significantly different between patients with and without steatosis (methotrexate 152.8 mg/kg, IQR 79.2–276.9 in steatosis patients vs 108.3 mg/kg, IQR 50.8–241.3 mg/kg in non-steatosis patients, *p* = 0.302, and steroids 36.7 mg/kg, IQR 0–951.9 in steatosis patients vs. 60.5 mg/kg, IQR 0–271.3 in non-steatosis patients, *p* = 0.184). The durations of methotrexate and steroid usage were not significantly different between patients with and without steatosis. There was no significant difference in the percentages of concomitant hepatotoxic drug use, such as sulfasalazine, azathioprine, leflunomide, and biologic DMARDs, between patients with and without hepatic steatosis.

### Predictors of hepatic steatosis in patients with JIA during methotrexate treatment

After a comparison of the clinical characteristics and laboratory data between patients with and without steatosis, notable clinical parameters were included in a further analysis. Multivariable logistic regression analysis showed that JIA patients with a BMI *Z*-score > 1 (OR 5.71, 95%CI 1.31–24.98, *p* = 0.021) and 100 mg cumulative methotrexate dose (OR 1.02, 95%CI 1.00–1.04, *p* = 0.041) were more likely to have hepatic steatosis (Table 4).

**Table 4:** Predictors of hepatic steatosis in patients with JIA treated with methotrexate

Parameter	Univariable		Multivariable	
	OR (95%CI)	*p*-value	OR (95%CI)	*p*-value
BMI *Z*-score > 1	5.86 (1.46–23.44)	0.012	5.71 (1.31–24.98)	0.021
Disease duration	1.20 (1.00–1.43)	0.052		
Positive history of elevated transaminases	4.84 (1.00–24.26)	0.055		
Duration of methotrexate use	1.01 (1.00–1.03)	0.112		
100 mg of cumulative methotrexate dose	1.02 (1.00–1.04)	0.027	1.02 (1.00–1.04)	0.041
100 mg of cumulative steroids dose	1.00 (1.00–1.01)	0.097		
GGT	1.04 (1.00–1.08)	0.084		

*BMI* body mass index, *GGT* gamma-glutamyl transferase

### Assessment of patients with JIA who had a normal BMI Z-score and hepatic steatosis

The clinical characteristics and laboratory data of patients with a normal BMI *Z*-score were compared between those with (*n* = 7) or without (*n* = 41) hepatic steatosis (Table 5). The disease duration, degree of disease activity, serum ALT, and lipid profiles were not significantly different between the two groups. The median cumulative dose of methotrexate tended to be higher in patients with hepatic steatosis (4665 mg, IQR 2541–8196) compared with patients without hepatic steatosis (3593 mg, IQR 1489–5592) (*p* = 0.147). Although the BMI *Z*-score was within the normal range for both groups, the BMI *Z*-score was significantly higher in patients with steatosis (0.27, 0.07–0.86) than in patients without steatosis (− 0.49, − 1.04–0.22).

**Table 5:** Clinical data of JIA patients who had a normal BMI *Z*-score with and without hepatic steatosis

Parameter	Patients with normal BMI *Z*-score who had steatosis (*N* = 7)	Patients with normal BMI *Z*-score who did not have steatosis (*N* = 41)	*p*-value
Female, *n* (%)	3 (42.9)	26 (63.4)	0.412
Age (years)	14.2 (12.3–17.3)	12.5 (9.6–15.0)	0.070
Disease duration (years)	5.6 (3.2–7.0)	3.8 (1.8–7.3)	0.358
BMI *Z*-score	0.27 (0.07–0.86)	− 0.49 (− 1.04–0.22)	*0.035*
JIA subtypes, *n* (%)			0.206
Systemic arthritis	3 (42.9)	10 (24.4)	
Enthesitis-related arthritis	3 (42.9)	8 (19.5)	
Polyarticular RF +	0 (0)	12 (29.3)	
Polyarticular RF-	0 (0)	4 (9.8)	
Oligoarticular	1 (14.3)	6 (14.6)	
Undifferentiated	0 (0)	1 (2.4)	
JADAS-27 scores	4.1 (1.0–7.3)	6.0 (2.0–10.3)	0.328
Medication information			
Duration of methotrexate use (months)	52.3 (35.2–83.0)	43.0 (17.9–82.7)	0.422
Cumulative dose of methotrexate (mg)	4665 (2541–8196)	3593 (1489–5592)	0.147
Duration of steroid use (months)	7.2 (0–49)	0 (0–19)	0.567
Cumulative dose of steroid (mg)	1733 (0–24,465)	0 (0–6114)	0.405
Concomitant use of leflunomide, *n* (%)	1 (14.3)	5 (12.2)	1.000
Concomitant use of anti-TNF agents, *n* (%)	2 (28.6)	8 (19.5)	0.549
Laboratory			
Maximum ALT (U/L)	40 (31–45)	26 (19–64)	0.439
Lipid profile level			
Cholesterol (mg/dL)	184 (147–239)	174 (157–202)	0.567
LDL cholesterol (mg/dL)	145 (91–168)	119 (90.5–143)	0.389
HDL cholesterol (mg/dL)	48 (43–60)	53 (45–69)	0.389
Triglyceride (mg/dL)	62 (45–103)	71 (54–103)	0.548

*ALT* alanine transaminase, *BMI* body mass index, *HDL* high-density lipoprotein, *IQR* interquartile range, *JADAS* Juvenile Arthritis Disease Activity Score, *JIA* juvenile idiopathic arthritis, *LDL* low-density lipoprotein, *RF* rheumatoid factor, *TNF* tumor necrosis factor

The details of seven JIA patients with a normal BMI *Z*-score and hepatic steatosis are shown in Table 6. All these patients had high disease activity except for patient 7 who had the longest duration of methotrexate use and the highest cumulative dose of methotrexate. Among these seven patients, the duration of methotrexate usage was > 2 years, and the cumulative dose of methotrexate was > 2300 mg. Four patients (patients 1, 2, 6, and 7) had abnormal levels of LDL, but none of them received lipid-lowering drugs.

**Table 6:** Summary of details from patients with JIA who had normal weight and exhibited hepatic steatosis

Patient	Age/sex	JIA type	Disease duration (years)	JADAS-27	MTX duration (months)	MTX CD (mg)	Steroid duration (months)	Steroid CD (mg)	Ever had abnormal ALT	Abnormal LDL
1	16.4/F	SJIA	7.0	22.5	83.0	8196	57.1	24,465	Yes	Yes
2	14.2/M	SJIA	6.6	25.5	76.0	6606	49	14,910	Yes	Yes
3	11.8/F	SJIA	5.6	23.2	35.2	2315	33.9	28,849	Yes	No
4	13.9/M	ERA	2.0	21	24.3	2451	7.2	1733	Yes	No
5	12.3/M	ERA	4.5	23.4	52.3	4665	0	0	No	No
6	17.3/M	ERA	3.2	11.4	37.2	3899	0	0	Yes	Yes
7	19.4/F	Oligo	12.8	4	151.0	13,929	0	0	Yes	Yes

*ALT* alanine transaminase, *CD* cumulative dose, *ERA* enthesitis related arthritis, *JADAS* Juvenile Arthritis Disease Activity Score, *JIA* juvenile idiopathic arthritis, *LDL* low-density lipoprotein, *MTX* methotrexate, *Oligo* oligoarticular, *SJIA* systemic juvenile idiopathic arthritis

## Discussion

To the best of our knowledge, this cross-sectional study is the first to evaluate methotrexate-associated hepatic complications, including hepatic fibrosis and hepatic steatosis in patients with JIA who received the long-term administration of methotrexate using TE. Among patients with a median methotrexate usage of 45 months and a median cumulative methotrexate dose of 3768 mg, none had hepatic fibrosis, and 21.7% had hepatic steatosis. Additionally, we revealed that a BMI *Z*-score > 1 and a cumulative dose of methotrexate were potential predictors of hepatic steatosis.

Methotrexate is a folate antagonist that inhibits several enzymes in the folate pathway. Its anti-inflammatory effect is mediated by adenosine accumulation, the inhibition of purine and pyrimidine syntheses, and inhibition of cell-mediated immunity [21]. Long-term methotrexate usage is associated with the accumulation of methotrexate metabolites and increase in oxidative stress in the liver, resulting in inflammation and fibrosis. The elevation of serum transaminases is quite common in patients with JIA treated with methotrexate, occurring in about 14–50% of cases [22–25]. This study found that 60% of patients had a history of elevated transaminases, and 13% had serum transaminases > 3 times the ULN. The variation in the frequency of elevated serum transaminases between studies might be related to the differences in the cutoff values used for transaminases. A study by Hashkes et al. [25] defined any elevation of serum transaminases beyond the ULN as abnormal, whereas other studies used values of transaminases > 2 times the ULN [23, 24]. Moreover, a previous study showed that combination therapy with methotrexate and leflunomide increased the risk of elevated transaminases in RA patients [26]. Therefore, patients with JIA who had used hepatotoxic medications, such as NSAIDs, sulfasalazine, and leflunomide, were excluded from a study that reported a low incidence of abnormal serum transaminases [24].

The prevalence of TE-defined hepatic fibrosis in RA patients on long-term methotrexate treatment was 3–27% [20, 27, 28]. Predictors for hepatic fibrosis in adult RA patients treated with methotrexate were old age, high cumulative dose of methotrexate, long disease duration, high BMI, and fatty liver disease. However, other studies demonstrated that methotrexate usage did not increase the risk for fibrosis [29, 30]. Data on the prevalence of hepatic fibrosis in JIA patients are very limited. Two studies evaluating the liver elasticity in JIA patients receiving methotrexate using shear-wave elastography reported discrepant results [24, 31]. In one study, the liver stiffness increased in 25 JIA patients compared with 25 healthy controls, whereas the other study showed no difference in liver stiffness between 49 JIA patients and 48 controls [24, 31]. In a study by Güngörer et al., the weekly methotrexate dose (*r*^2^ = 0.502, *p* = 0.011) and age (*r*^2^ = 0.491, *p* = 0.03) correlated with liver shear-wave elastography values but not the cumulative dose of methotrexate [31]. However, regression analysis was not used to confirm the association between the weekly methotrexate dose and liver elasticity in that study. In our study, only age correlated positively with the LSM value, whereas the cumulative dose and weekly dose of methotrexate were not associated with the LSM. Previous studies demonstrated that liver stiffness increased with age using magnetic resonance elastography and TE [32, 33]. A study by Hashkes et al. reported mild hepatic fibrosis in 15% of 25 patients with JIA treated with methotrexate who underwent liver biopsy and indicated the BMI was a risk factor for fibrosis [25]. In contrast to our study, hepatic fibrosis was not found in any JIA patients whose median duration of methotrexate usage was 45 months. The median LSM value in our study was similar to that of healthy children in previous studies [32, 34]. This contrary result might be explained by the higher BMI of patients in the study by Hashkes et al. (mean BMI 22.5 kg/m^2^, SD 5.3) compared with our patients (mean BMI 16.3 kg/m^2^, SD 2.7) with a longer disease duration (mean 9.8 years, SD 3.9). Although TE had a high sensitivity (90%) and specificity (90%) for the detection of significant fibrosis in patients with newly diagnosed liver disease, it had lower sensitivity (77%) and specificity (67%) for the detection of mild fibrosis [35]. However, Lahdenne et al. evaluated hepatic fibrosis in 34 JIA patients on methotrexate for more than 2.4 years using histopathological findings and did not find any cases of hepatic fibrosis, similar to our study [36]. Our study provided evidence that long-term methotrexate usage and the use of methotrexate in combination with other DMARDs including leflunomide did not increase the risk for TE-defined hepatic fibrosis in JIA patients. Also, all patients in this study received folic acid supplementation as a previous study showed its hepatoprotective effect [37]. Recent strategies in our study including serial monitoring of liver chemistry, timely drug adjustments (if necessary), and folic acid supplementation might prevent the development of hepatic injury [37].

The mechanism involved in how methotrexate is involved in hepatic steatosis remains unclear but might be associated with folate antagonism, deficiency of purines, pyrimidine thymidine, and accumulation of methotrexate polyglutamates in hepatic tissues [38]. At the initial stage, methotrexate causes hepatic steatosis, which is reversible. Later, fibrosis and cirrhosis occur if there is no intervention such as drug cessation or dose reduction. Steatosis was the most prevalent histological finding in liver biopsy samples from RA and JIA patients treated with methotrexate [36, 39]. The prevalence of hepatic steatosis in RA patients treated with methotrexate was 5–39% [39, 40]. These varied results might be explained by differences in diagnostic techniques and the criteria used for classifying steatosis. A meta-analysis of pooled data using various techniques including liver enzyme tests, imaging, and biopsy evaluated the prevalence of hepatic steatosis in children as 7.6% [41]. Of note, the prevalence differed by ethnicity. Asian children (10.2%) had a higher prevalence than white (non-Hispanic) children (8.6%) [42]. In this study, hepatic steatosis was identified in 21.7% of our JIA patients, which was higher than that in the general pediatric population.

Previous studies of RA patients demonstrated that a high BMI, diabetes mellitus, hypertriglyceridemia, hypercholesterolemia, and cumulative dose of methotrexate were risk factors for hepatic steatosis [39, 40, 43]. Obesity and hyperlipidemia are components of metabolic syndrome and are recognized as predictors associated with non-alcoholic fatty liver disease [44]. This study showed that serum cholesterol and triglyceride levels were not different between patients with JIA with or without hepatic steatosis, which is in contrast to previous studies of RA patients [39, 40]. With advancing age, the prevalence of metabolic syndrome including hypertension, diabetes mellitus, and hyperlipidemia increases considerably, which might explain why these factors are independent predictors of hepatic steatosis in adult patients with RA. In this study, a BMI *Z*-score > 1 was a potential predictor of hepatic steatosis in JIA patients treated with methotrexate. A study of RA patients by Gremese et al. reported a link between a high BMI and worse disease activity and refractory disease, resulting in persistent systemic inflammation and increased risk for hepatic steatosis [45]. Therefore, lifestyle modification may reduce hepatic steatosis and achieve disease control. Erre et al. and Sakthiswary et al. reported that the mean cumulative dose of methotrexate was associated with hepatic steatosis in RA patients [40, 43], which was similar to our study. However, TE to evaluate the pre-existing hepatic steatosis was not performed in our patients before starting methotrexate. Hepatic steatosis in methotrexate-treated patients might be a result of being overweight/obese rather than a primary effect of methotrexate. However, JIA patients with a normal BMI *Z*-score and hepatic steatosis tended to have a higher cumulative dose of methotrexate than those with a normal BMI *Z*-score but no hepatic steatosis. To confirm the cause and effect of independent predictors, a long-term prospective study excluding potential risk factors that contribute to hepatic steatosis should be initiated to assess methotrexate-induced hepatic injury.

Interestingly, about two third of JIA patients with TE-defined hepatic steatosis had serum AST and ALT levels within the normal range. Therefore, the usage of ALT and AST levels alone was insufficient to detect hepatic steatosis. A previous study of JIA patients treated with methotrexate suggested that serial liver enzyme abnormalities were related to histopathologic abnormalities [25]. In contrast with our study, the frequency of elevated transaminases was not associated with the occurrence of steatosis.

This study had several limitations. First, the small sample size resulted in insufficient power to robustly evaluate uncommon hepatic-related complications including hepatic fibrosis in JIA patients treated with methotrexate. Also, the limited number of patients might overestimate the effect measure of predictors. Second, patients diagnosed with hepatic steatosis were not confirmed by liver biopsy, which might provide a more accurate estimate of the prevalence of hepatic steatosis.

## Conclusions

Long-term low-dose methotrexate usage for nearly 4 years and the concomitant use of other DMARDs did not increase the risk of TE-defined hepatic fibrosis in JIA patients. However, hepatic steatosis was common among patients with JIA receiving methotrexate. Overweight/obese JIA adolescents and patients with a higher cumulative dose of methotrexate were at risk for hepatic steatosis. Because hepatic steatosis is clinically silent, TE should be considered in high-risk patients during methotrexate treatment to prevent irreversible hepatic-related complications.

## Data Availability

The datasets generated during and/or analyzed during the current study are available from the corresponding author on reasonable request.

## Abbreviations

*ALT*: Alanine transaminase
*AST*: Aspartate transaminase
*BMI*: Body mass index
*CAP*: Controlled attenuation parameter
*CI*: Confidence interval
*dB/m*: Decibel/meter
*DMARDs*: Disease-modifying antirheumatic drugs
*ESR*: Erythrocyte sedimentation rate
*HDL*: High-density lipoprotein
*IQR*: Interquartile range
*JADAS*: Juvenile Arthritis Disease Activity Score
*JIA*: Juvenile idiopathic arthritis
*kPa*: Kilopascal
*LDL*: Low-density lipoprotein
*LSM*: Liver stiffness measurement
*NSAIDs*: Nonsteroidal anti-inflammatory drugs
*OR*: Odds ratio
*RA*: Rheumatoid arthritis
*TE*: Transient elastography
*ULN*: Upper limit of normal
